# Prognostic significance of LRRC1 in hepatocellular carcinoma and construction of relevant prognostic model

**DOI:** 10.1097/MD.0000000000034365

**Published:** 2023-07-28

**Authors:** Qingshan Cai, Dongyang Wu, Yueling Shen, Shudong Li, Liyou Liu, Dong Liu, Yong Li, Xiaonan Chen, Limin Wang, Jianxing Zheng

**Affiliations:** a Department of Hepatobiliary Surgery, Tangshan Central Hospital, Hebei Province, China; b Department of Otolaryngology, Qianan People’s Hospital, Hebei Province, China; c Department of General Surgery, Tangshan Eighth Hospital, Hebei Province, China; d Hepatobiliary Surgery Department, Tangshan Gongren Hospital, Hebei Province, China.

**Keywords:** hepatocellular carcinoma, immune, LRRC1, prognosis

## Abstract

This study aimed to elucidate the prognostic value of the leucine rich repeat containing 1 (LRRC1) gene in hepatocellular carcinoma (HCC) and to determine the effects of high and low LRRC1 expression on mutation and immune cell infiltration. We downloaded HCC mRNA-seq expression and clinical data from University of California Santa Cruz Xena. The expression of LRRC1 was compared between HCC tumor and normal samples. Tumor samples were divided according to high and low LRRC1 expression. Differentially expressed genes between the 2 groups were identified, and function, mutation, and immune cell infiltration were analyzed. Genes associated with immune cells were identified using weighted gene co-expression network analysis, and transcription factors of these genes were predicted. Moreover, a prognostic model was developed and its performance was evaluated. The expression of LRRC1 was upregulated in HCC tissues, and this indicated a poor prognosis for patients with HCC. Differentially expressed genes between high and low LRRC1 expression were significantly enriched in pathways associated with cancer, amino acid metabolism, carbohydrate metabolism, and the immune system. We identified 15 differentially infiltrated immune cells between tumors with high and low LRRC1 expression and 14 of them correlated with *LRRC1* gene expression. Weighted gene co-expression network analysis identified 83 immune cell-related genes, 27 of which had prognostic value. Cyclic AMP-response element binding protein regulated annexin A5, matrix metallopeptidase 9, and LRRC1 in the transcription factor regulatory network. Finally, a prognostic model composed of 7 genes were generated, which could accurately predict the prognosis of HCC patients. The LRRC1 gene might serve as a potential immune-associated prognostic biomarker for HCC.

**Key points**

1. Upregulated LRRC1 indicated poor prognosis for patients with HCC.
2. Expression of LRRC1 correlated with infiltration of macrophages, Tregs, and NK cells.
3. Different LRRC1 expression profiles altered mutation landscape.
4. CREB1 regulated ANXA5, MMP9, and LRRC1 in a TF regulatory network.
5. A 7-gene signature could well classify HCC samples into low/high risk groups.

## 1. Introduction

Liver cancer is the third leading cause of cancer-related deaths globally, with 83% of the cases present in less developed regions.^[1,2]^ Hepatocellular carcinoma (HCC) accounts for ~90% of primary liver malignancies, and rapid growth, early hepatic metastasis, and rapid multidrug resistance leads to a poor prognosis.^[3]^ Global estimates indicate that China will have the highest prevalence of HCC among all countries by 2030.^[4]^ HCC remains a global clinical challenge because of its high incidence, limited therapeutic strategies, and poor prognosis.^[5]^ Therefore, identifying useful prognostic biomarkers and/or therapeutic targets for HCC might help to improve the prognosis of patients with HCC.

In recent years, with the rapid development of gene sequencing technology, our understanding of the molecular pathogenesis of HCC has been significantly improved. In particular, high-throughput sequencing data can be used to identify key biomarkers associated with HCC progression, and these specific gene expression signature can effectively predict the risk and prognosis of HCC, providing new options for treatment.^[6,7]^ For example, Chen et al found that high expression of MCM10 was associated with poor prognosis of patients with HCC and was an independent prognostic indicator for HCC^[8]^; Ma et al showed that high expression of H2AFY predicted poor prognosis in HCC patients^[9]^; Huo et al revealed that CTNNB1 mutations-related metabolic prognostic markers could be used in clinical practice to determine the prognosis of individual with HCC.^[10]^ Taken together, biomarkers are relatively simple and noninvasive approach to detect and estimate disease prognosis, and will help physicians to better monitor patient outcomes and improve overall survival. Despite efforts to identify molecules as potential biomarkers, the number of available biomarkers known to be associated with the prognosis of HCC remains limited.

Leucine-rich repeat containing 1 (LRRC1; also known as LANO), is a 524 amino acid protein in the leucine-rich repeat (LAP) and post-synaptic density protein-95, Drosophila disc large tumor suppressor, and Zonula occludens-1 protein (PDZ) protein families.^[11]^ The LAP protein family in mammals includes SCRIB (the core component of the Scribble complex), Erbb2 interacting protein (ERBIN), Densin-180 (also known as LAP-1 and LAP) and LRRC1.^[12]^ Scribble is 1 of 3 types of polar complexes that significantly affect the apical-basal polarity of epithelial cells.^[13]^ Most human cancers, including HCC, originate from epithelial tissues, and a loss of cell polarity and tissue structure is often a hallmark of such malignancies.^[11,14]^ Therefore, aberrant expression of SCRIB is frequently observed in several human cancers, including prostate, colorectal, and liver cancer.^[15–18]^ The paralog of mammalian SCRIB, LRRC1, shares ~60% identity with human SCRIB,^[19]^ but its role in cancer remains largely elusive. Although LRRC1 promotes HCC cell proliferation,^[11,20]^ whether LRRC1 affects the prognosis of HCC has not been elucidated in detail.

Therefore, this study aimed to elucidate the prognostic value of the LRRC1 gene in HCC and to determine the effects of its high or low expression on mutation and immune cell infiltration. We also analyzed genes associated with immune cells in HCC based on LRRC1 gene expression to identify potential prognostic markers associated with HCC. Further, a prognostic model for HCC based on these prognostic markers was established and validated.

## 2. Materials and methods

### 2.1. Data sources

Information about HCC mRNA-seq expression (normalized log_2_; FPKM + 1) and clinical details was downloaded from the University of California Santa Cruz Xena browser (https://xena.ucsc.edu/),^[21]^ and the detection platform was an Illumina HiSeq 2000 RNA sequencing system. The mRNA-seq included 424 samples, including 50 normal, and 374 tumor samples. Mutation data were downloaded from the cancer genome atlas (TCGA). Moreover, the RNA-seq data of 221 HCC patients and their clinical parameters were extracted from the NCBI GEO database (ID: GSE14520).

### 2.2. Expression and prognostic value of LRRC1 in HCC

We compared LRRC1 expression between HCC tumor and normal samples using the inter-group Wilcox test in R4.1.1. Then, based on the clinical prognosis information and the expression level of LRRC1, samples were classified into the LRRC1-high and LRRC1-low expression groups according to the median cutoff value. The survival of patients with HCC and high or low LRRC1 expression was analyzed using the GEPIA database.^[22]^ Levels of LRRC1 expression at various stages and grades were analyzed using the UALCAN database.^[23]^ Gene mutations were analyzed using the cbioportal database.^[24]^ Expression of the LRRC1 gene at the protein level was validated using the human protein atlas.^[25]^

### 2.3. Correlations between LRRC1 and clinical features

Correlations between LRRC1 and HCC clinical features (stage, grade, pathological N/T/M stage, age, gender, and race) were analyzed using inter-group Chi-square tests in R 4.1.1.

### 2.4. Gene differences and functional analysis of differentially expressed genes (DEGs) between the LRRC1 expression groups

We analyzed DEGs in samples from the TCGA-HCC dataset with high and low LRRC1 expression. We screened DEGs using the Limma package^[26]^ in R using the following thresholds: |log fold change (FC)| > 0.585 (FC > 1.5) and *P* < .05. Gene ontology (GO) and Kyoto encyclopedia of genes and genomes functional enrichment analyses of DEGs were performed using the R clusterProfiler package in R.^[27]^

### 2.5. Mutation landscape according to LRRC1 expression

Based on the mutation spectrum of HCC in TCGA, we calculated the mutation rates of genes under different LRRC1 expression patterns using the maftools v. 2.8.05 package in R.^[28]^ We then selected the top 20 genes with the highest mutation rate.

### 2.6. Analysis of tumor immune cell infiltration

We used the R GSVA package v. 1.42.0 in R for single-sample gene set enrichment analysis.^[29]^ We then analyzed difference in immune cell infiltration between the 2 LRCC1 expression groups. Additionally, correlation analysis was performed for LRRC1 gene and differentially infiltrated immune cells.

### 2.7. Key module screening using WGCNA

We screened module genes that were significantly associated with immunity based on differential and significantly associated immune cells as traits using the weighted gene co-expression network analysis (WGCNA) package v. 1.61 in R.^[30]^ Intersections of key module genes and DEGs were regarded as hub genes.

### 2.8. Prognosis and correlations of key module gene

Tumor samples were separated according to key gene expression above (high) and below (low) the median. We then analyzed the clinical prognoses and survival of patients with HCC using Kaplan–Meier [KM]) curves in Survival v. 2.41-1^[31]^ to screen out key genes related to prognosis. We then analyzed correlations between these genes and LRRC1 expression.

### 2.9. Analysis of regulatory factors of key prognostic genes

Because gene expression is regulated by transcription factors (TFs), we analyzed TFs regulate candidate key genes using the transcription factor database hTFtarget.^[32]^ The regulatory miRNAs of key genes were predicted using miRWalk.^[33]^ Regulatory pairs predicted by both TargetScan and miRDB in miRWalk were retained as the final targeted regulatory pairs. Finally, the miRNA-TF-mRNA regulatory network was constructed using Cytoscape.^[34]^

### 2.10. Prognostic model construction and validation

In the TCGA cohort, based on the expression levels of 27 key prognostic genes and LRRC1 in the HCC samples, a univariate Cox regression analysis was employed to identify genes that significantly correlated with overall survival, and the cutoff value set at *P* < .05. Afterward, the Lasso Cox regression algorithm and “glmnet” R package were used to eliminate redundancy, and the optimal prognostic signature was created using 20-fold cross-validation.^[35,36]^ The formula for risk score (RS) calculation was as follows: RS = ∑βgene × Exp gene (βgene and Exp gene represent the risk coefficient and the expression level of each gene, respectively). According to the median RS value, patients in the TCGA (training set) and GSE14520 (validation set) were divided into low risk (LR) and high risk (HR) groups. The KM method from the “survival” package v. 2.41-1^[31]^ was carried out to assess the association between different risk groups and survival prognosis. Moreover, the prognostic accuracy of the model in the training and validation sets was measured using the “timeROC” package.^[37]^

## 3. Results

### 3.1. Upregulated LRRC1 expression in HCC tumors

**Figure 1.**
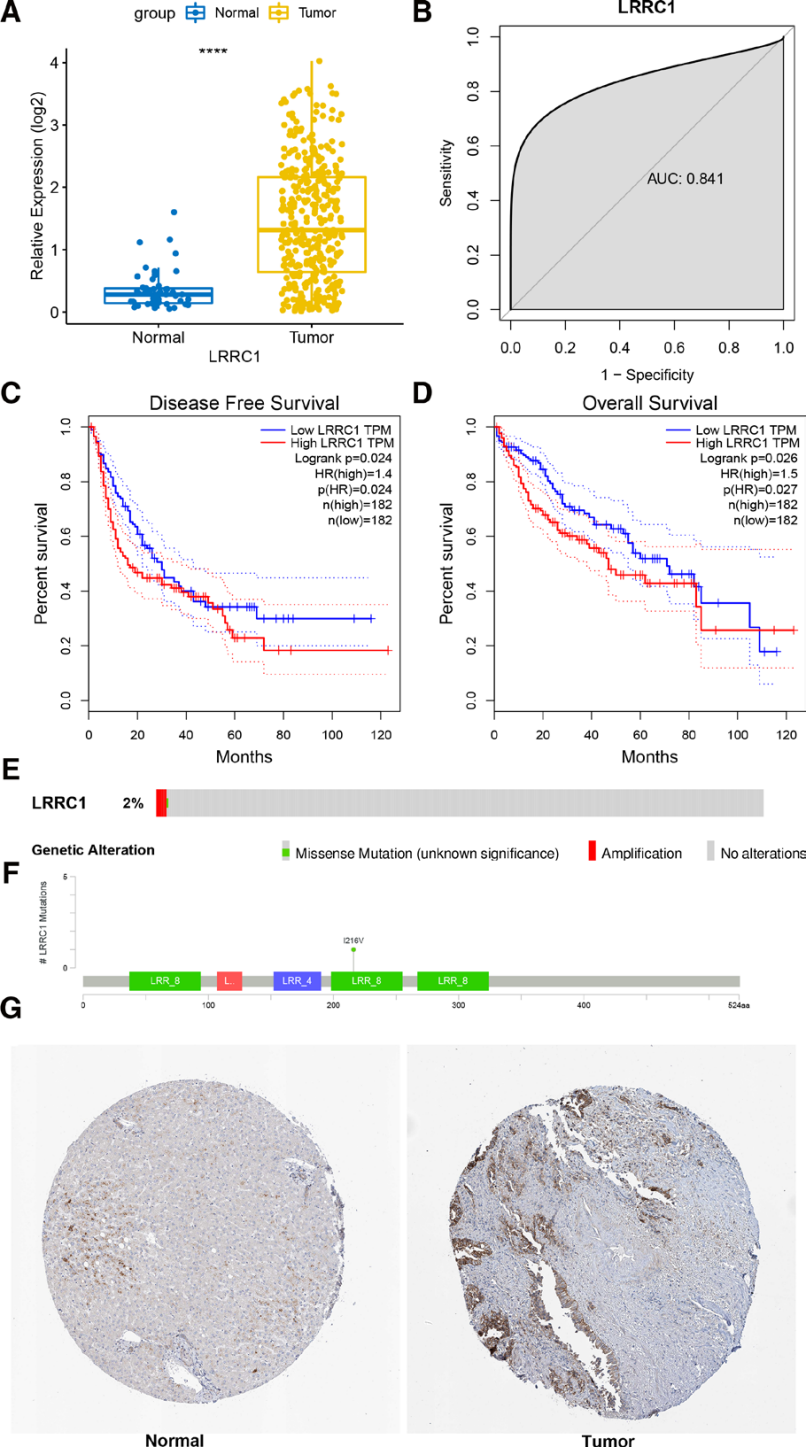
Expression of LRRC1 in HCC, ROC curves, survival rates, mutations and immunohistochemical findings of LRRC1. (A) Expression of LRRC1 in HCC tumors and normal control tissues. (B) Diagnostic ROC curve of LRRC1 for HCC. Kaplan–Meier DFS (C) and OS (D) curves of patients with HCC with high and low LRRC1 expression. Mutation rates (E) and sites (F) of LRRC1 in HCC. (G) Immunohistochemical findings of LRRC1 in normal and HCC tissues. HCC = hepatocellular carcinoma, LRRC1 = leucine rich repeat containing 1, ROC = receiver operator characteristics.

The expression of LRRC1 in HCC tumors and normal controls were compared using inter-group Wilcox tests. Figure 1A shows significantly upregulated LRRC1 expression in HCC tumors. The diagnostic value of LRRC1 for HCC, evaluated based on the area under the receiver operator characteristics (ROC) curve (AUC), was 0.841, indicating a high diagnostic value (Fig. 1B). The survival of patients with HCC with high or low LRRC1 expression was investigated, and the results showed that low LRRC1 expression indicated good disease-free and overall survival prognoses (Fig. 1C and D). Mutation rates and locations of LRRC1 are shown in Figure 1E and F.

### 3.2. LRRC1 protein was overexpressed in HCC tissue

We analyzed LRRC1 protein levels in normal and HCC tumor tissues using the human protein atlas database to further confirm that the prognostic value of LRRC1 is reliable. The results showed that LRRC1 was overexpressed in tumor, compared to normal tissues, which was consistent with the results at the transcription level (Fig. 1G).

### 3.3. Relationship between LRRC1 and clinical features

**Figure 2.**
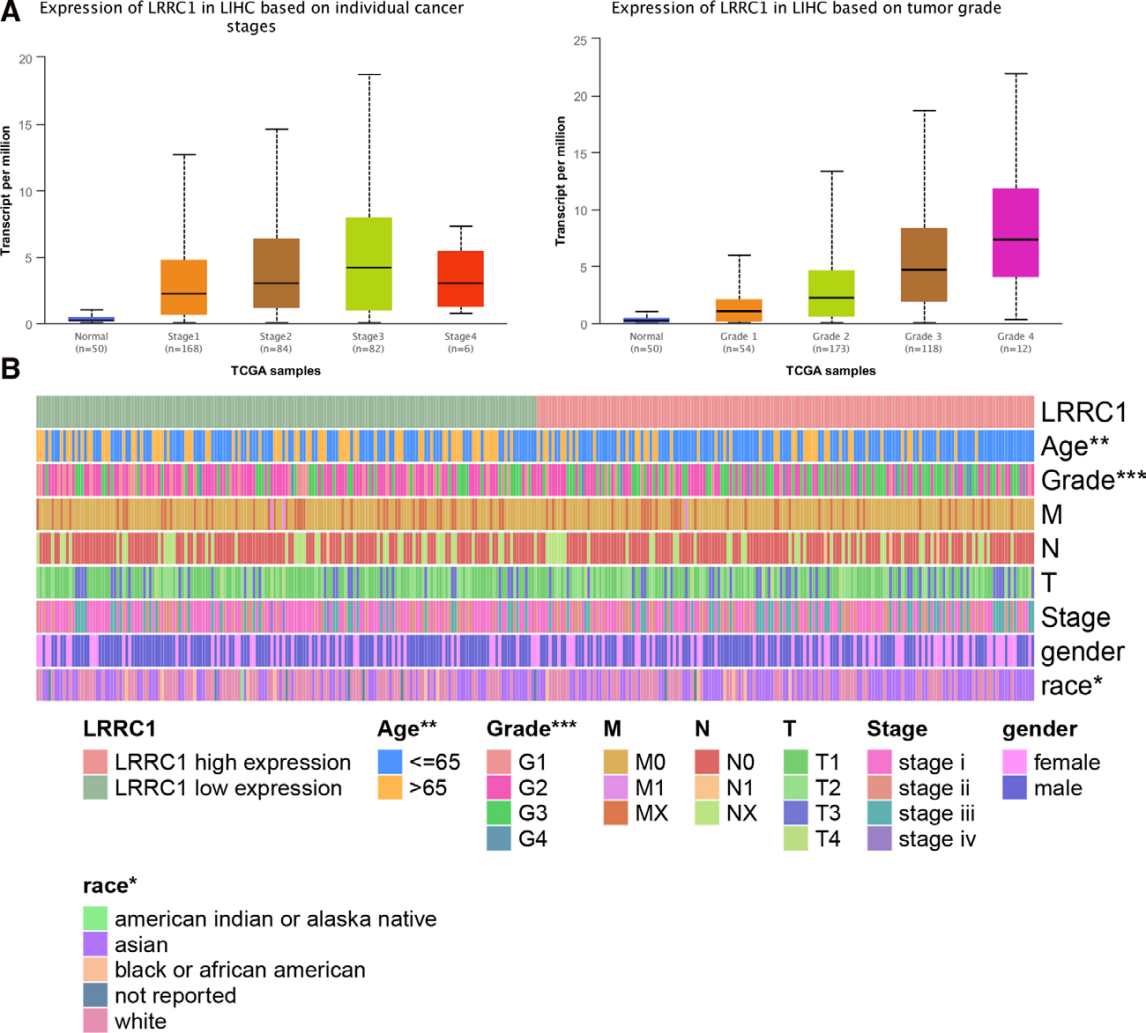
Expression of LRRC1 according to tumor status and heatmap of LRRC1 and clinical features. (A) Expression of LRRC1 differs according to clinical stage and tumor grade. (B) Heatmap shows correlations between LRRC1 and clinical features. LRRC1 = leucine rich repeat containing 1.

We analyzed LRRC1 expression at different clinical stages. Tumors with high LRRC1 expression were associated with a poor clinical prognosis, indicating that LRRC1 is a tumor risk factor (Fig. 2A). Figure 2B shows correlations between LRRC1 expression and the histological stage, pathologic stage, and neoplasm grade of HCC tumors.

### 3.4. DEGs between LRRC1-high and -low expression groups and their functional analysis

**Figure 3.**
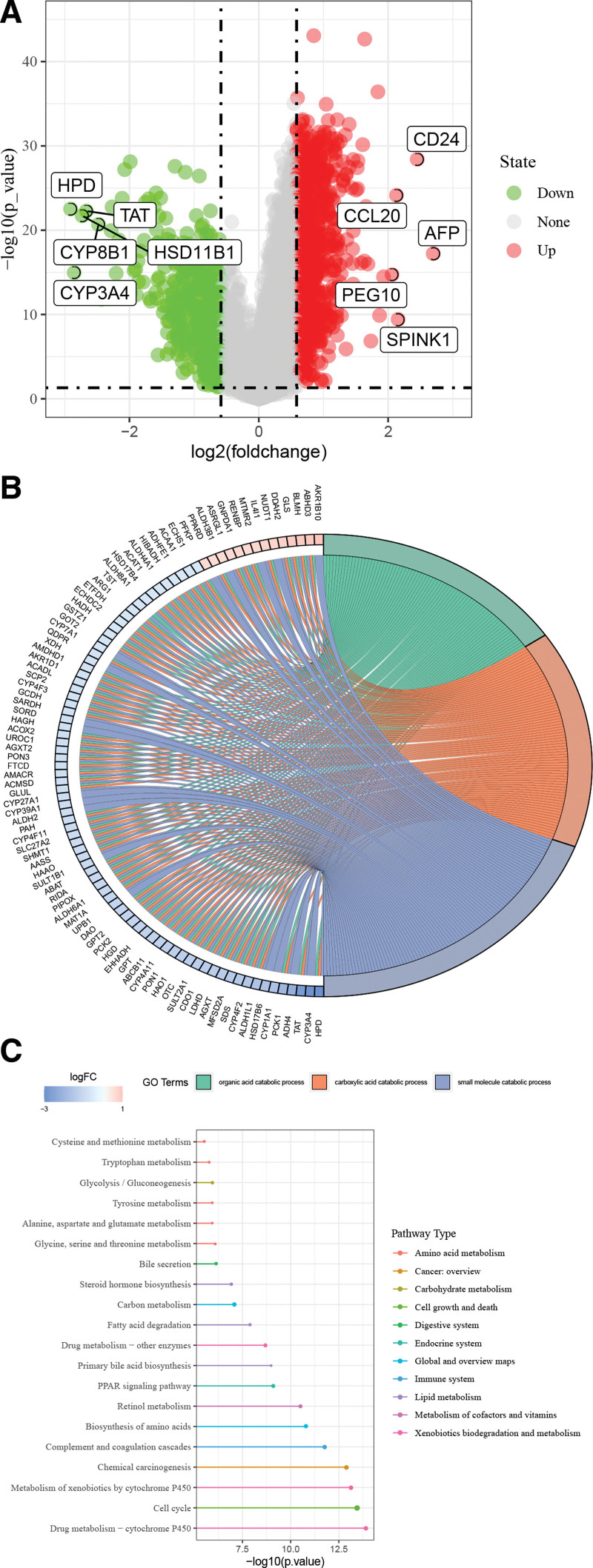
Differentially expressed genes in HCC under high and low LRRC1 expression and enriched GO terms and KEGG pathways. (A) Differentially expressed genes (DEGs) under high and low LRRC1 expression. Dots represent genes. Red and green dots represent significantly upregulated and downregulated DEGs, respectively. Enriched GO terms (B) and KEGG pathways (C) of DEGs in HCC with high and low LRRC1 expression. GO = gene ontology; HCC = hepatocellular carcinoma, KEGG = Kyoto encyclopedia of genes and genomes, LRRC1 = leucine rich repeat containing 1.

We identified 1360 DEGs (963 upregulated and 397 downregulated) between the LRRC1-high and -low groups (Fig. 3A). The top 3 GO terms enriched by these DEGs were organic acid, carboxylic acid, and small molecule catabolic processes (Fig. 3B). Figure 3C shows the top 20 pathways involving 11 types and including amino acid metabolism, cancer overview, carbohydrate metabolism, cell growth and death, and immune system.

### 3.5. Mutations under high and low LRRC1 expression

**Figure 4.**
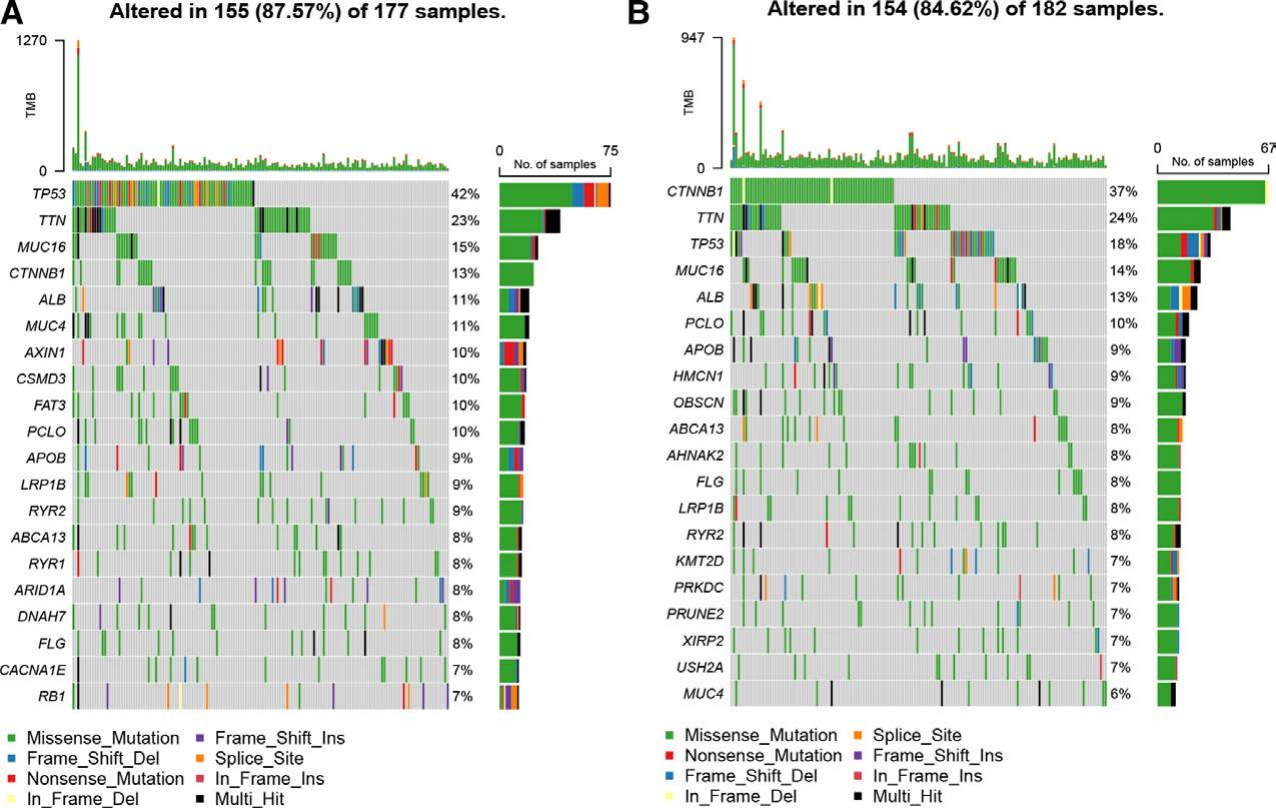
Waterfall diagram of mutations in LRRC1-high (A) and LRRC1-low (B) HCC tumors. HCC = hepatocellular carcinoma, LRRC1 = leucine rich repeat containing 1.

The mutation rates were significantly higher for the TP53 gene in the LRRC1-high compared to LRCC1-low group (Fig. 4A), and for the CTNNB1 gene in the LRRC1-low group compared to LRCC1-high group (Fig. 4B).

### 3.6. Tumor immune cell infiltration

**Figure 5.**
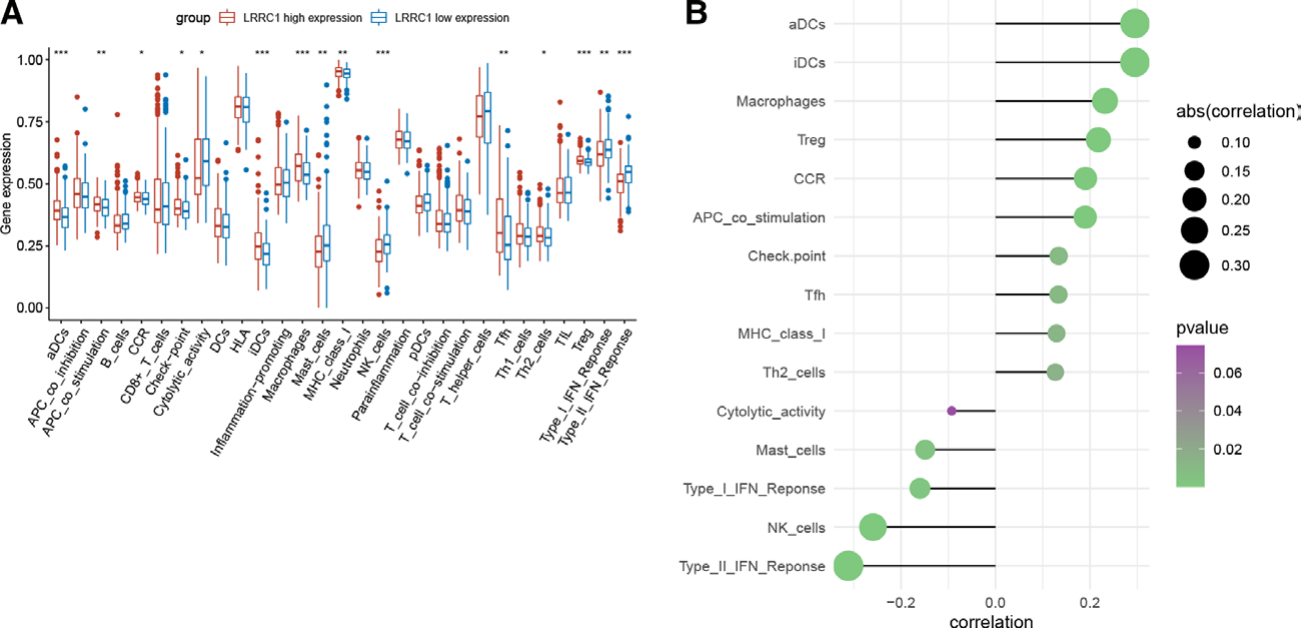
Differentially infiltrated immune cells and correlations with LRRC1. (A) Box plot of differentially infiltrated immune cells. (B) Correlations between LRRC1 and differentially infiltrated immune cells. LRRC1 = leucine rich repeat containing 1.

We identified 15 significantly differentially infiltrated immune cells between tumors with high and low LRCC1 expression (Fig. 5A). Among these cells, 14 significantly correlated with LRRC1 expression (Fig. 5B). For instance, LRCC1 correlated positively with aDCs, interdigitative DCs, macrophages, and regulatory T cells (Tregs), and negatively with cytolytic activity, mast cells, type I interferon response, and natural killer (NK) cells.

### 3.7. Screening key modules and genes related to immune cells using WGCNA

**Figure 6.**
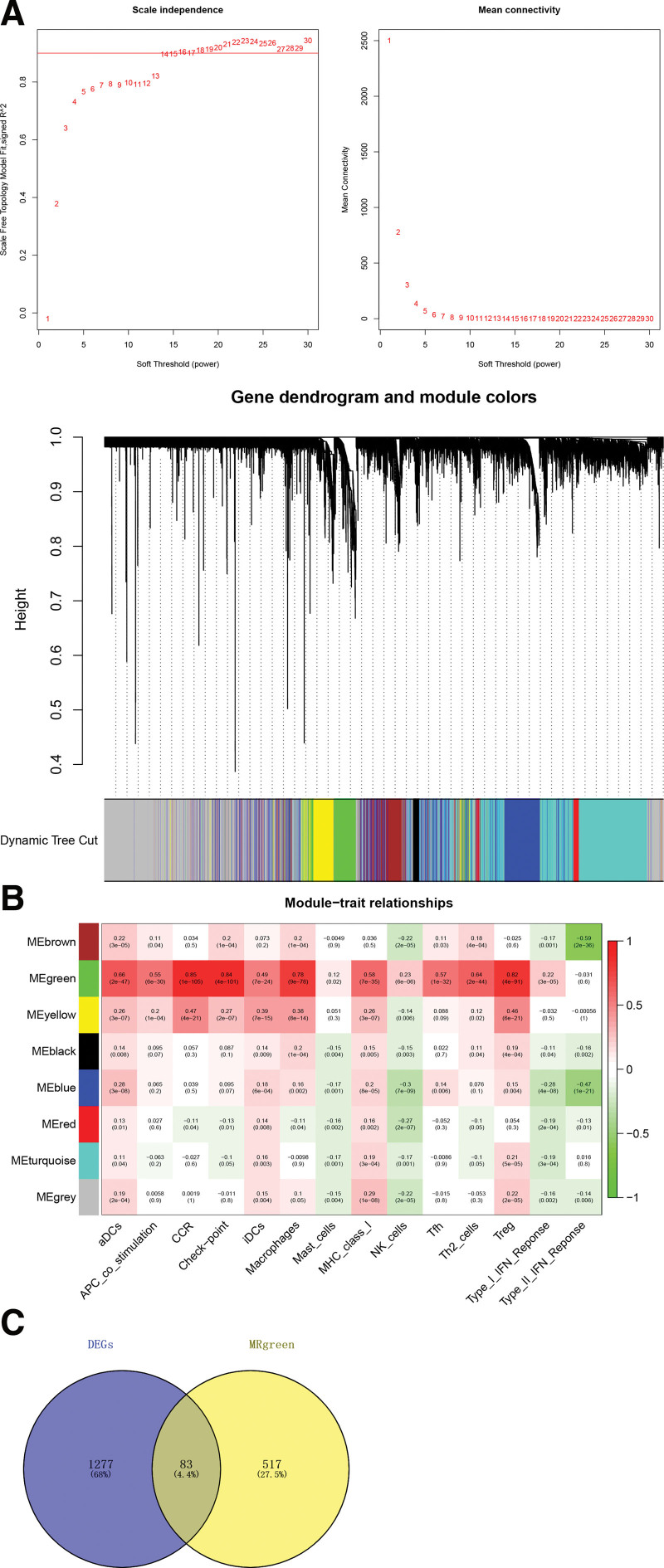
Hierarchical clustering of modules, their correlations with phenotype, and intersection of module genes and DEGs. (A) Modules divided by hierarchical clustering; different colors represent different modules. (B) Correlations between modules and phenotype (immune cells). (C) Venn diagram of intersection of module genes and differentially expressed genes (DEGs).

We constructed a co-expression network of genes with an average expression >0.5 using WGCNA. The power threshold was set at 14 (R^2^ = 0.9). The network was constructed using dynamic tree cutting to segment the modules (Fig. 6A). The modules were associated with phenotypes based on their correlations with sample traits. The correlation was the closest for the green module (Fig. 6B). Therefore, we identified it as the key module, which included 600 genes. These genes intersected with the 1360 DEGs, resulting in 83 intersection genes (Fig. 6C).

### 3.8. Prognostic analysis of key genes

**Figure 7.**
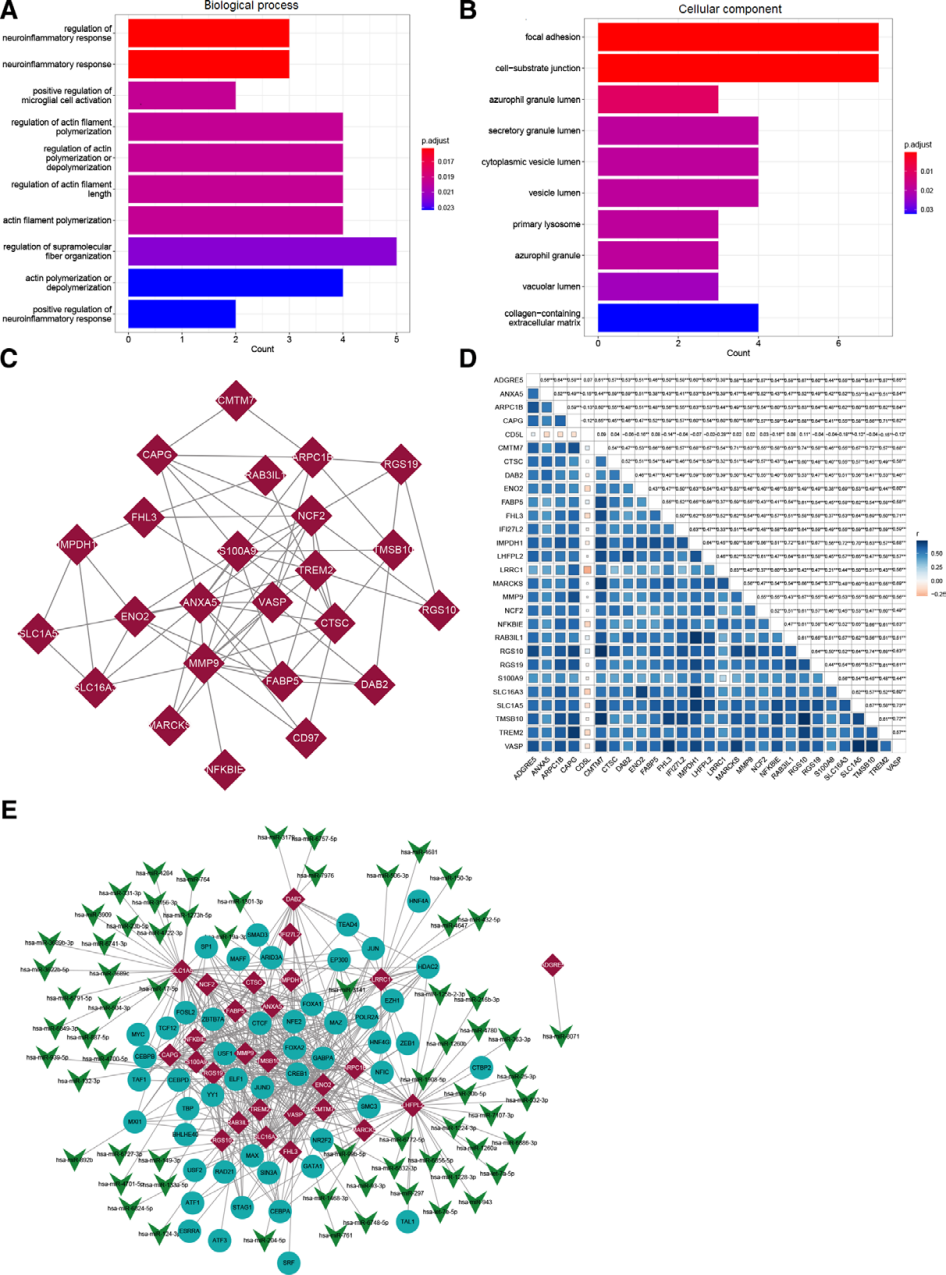
Functional enrichment, PPI network, correlations, and miRNA-TF-mRNA network. (A and B) Functional enrichment of GO biological process (A) and cellular components (B). (C) Protein-protein interaction network. (D) Correlations of key prognostic genes. (E) MicroRNA-TF-mRNA network. Red rhombus, mRNAs; blue circles, transcription factors (TF); green V-shapes miRNAs. GO = gene ontology; miRNA = micro ribonucleic acid, mRNA = messenger ribonucleic acid, PPI = protein-protein interaction.

KM survival curves showed that 27 of the 83 intersection genes were significantly associated with prognosis (Supplemental Figure S1, http://links.lww.com/MD/J321). We then analyzed the GO functions of these 27 genes and their protein-protein interaction (PPI) networks. Figure 7A and B shows that these genes were enriched in the GO biological processes of regulation of neuroinflammatory response, positive regulation of microglial cell activation, and regulation of actin filament polymerization, and the cellular components of focal adhesion, cell-substrate junction, and azurophil granule lumen. Figure 7C shows the PPI network. Annexin A5 (ANXA5), matrix metallopeptidase 9 (MMP9), and neutrophil cytosolic factor 2 (NCF2) were the top 3 hub nodes. We found that all key genes positively correlated with LRRC1 except the CD5 molecule like (CD5L) gene (Fig. 7D).

### 3.9. Regulatory network of key prognostic gene

The TFs that regulate LRRC1 and candidate key genes were analyzed using the transcription factor database hTFtarget, and we uncovered a relationship network of 50 TFs corresponding to 24 genes. In addition, 66 miRNAs were identified for 15 genes. Finally, a miRNA-TF-mRNA regulatory network was constructed (Fig. 7E). The degree of CAMP responsive element binding protein 1 (CREB1), which regulated the hub genes ANXA5, MMP9, and LRRC1, was the highest among the TFs in the network. We found that LRRC1 was regulated by 5 miRNAs, including miR-150-3p.

### 3.10. Identification and construction of prognostic signature

**Figure 8.**
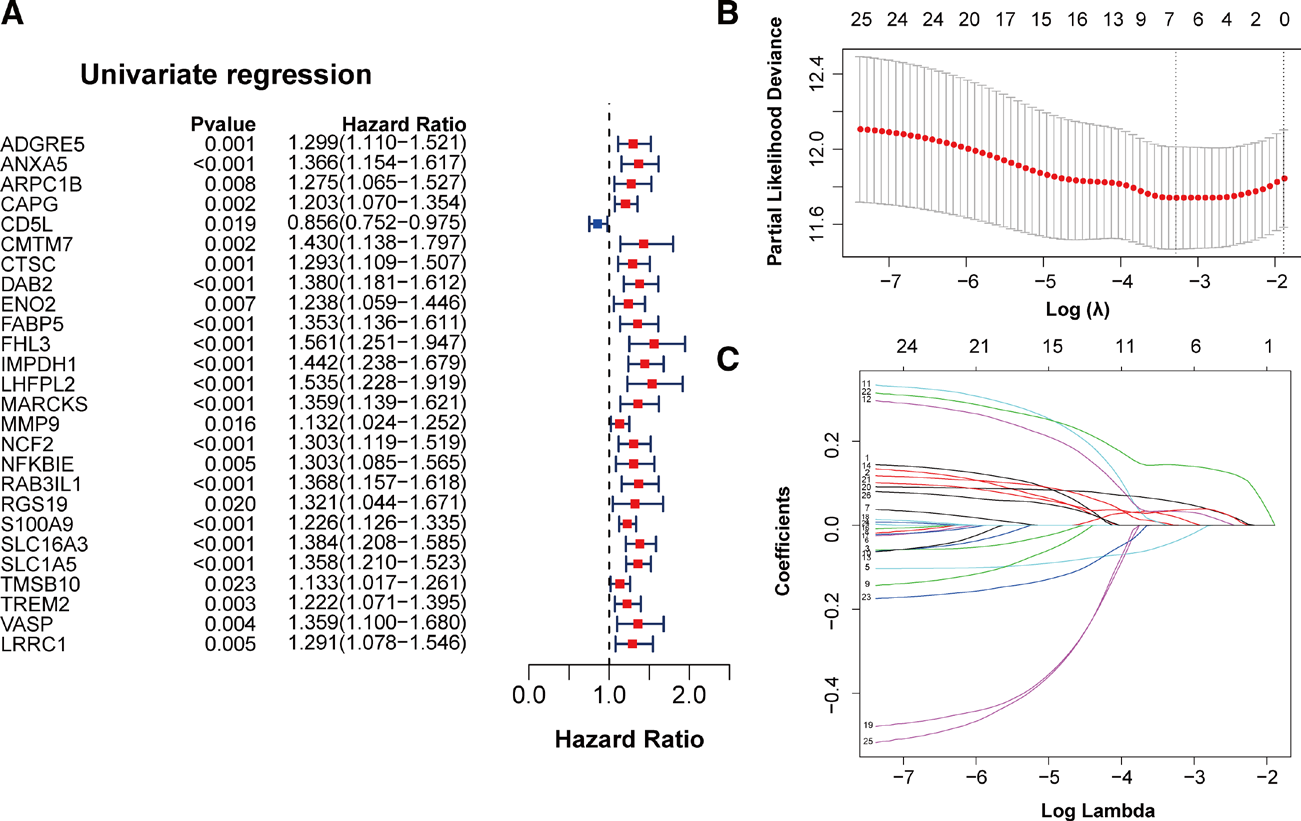
Construction of prognostic model. (A) Univariate Cox regression analysis of overall survival-related genes (*P* < .05). (B and C) Lasso Cox regression analysis shows the optimal coefficients (B) and minimum lambda (C) of the survival-related genes.

**Figure 9.**
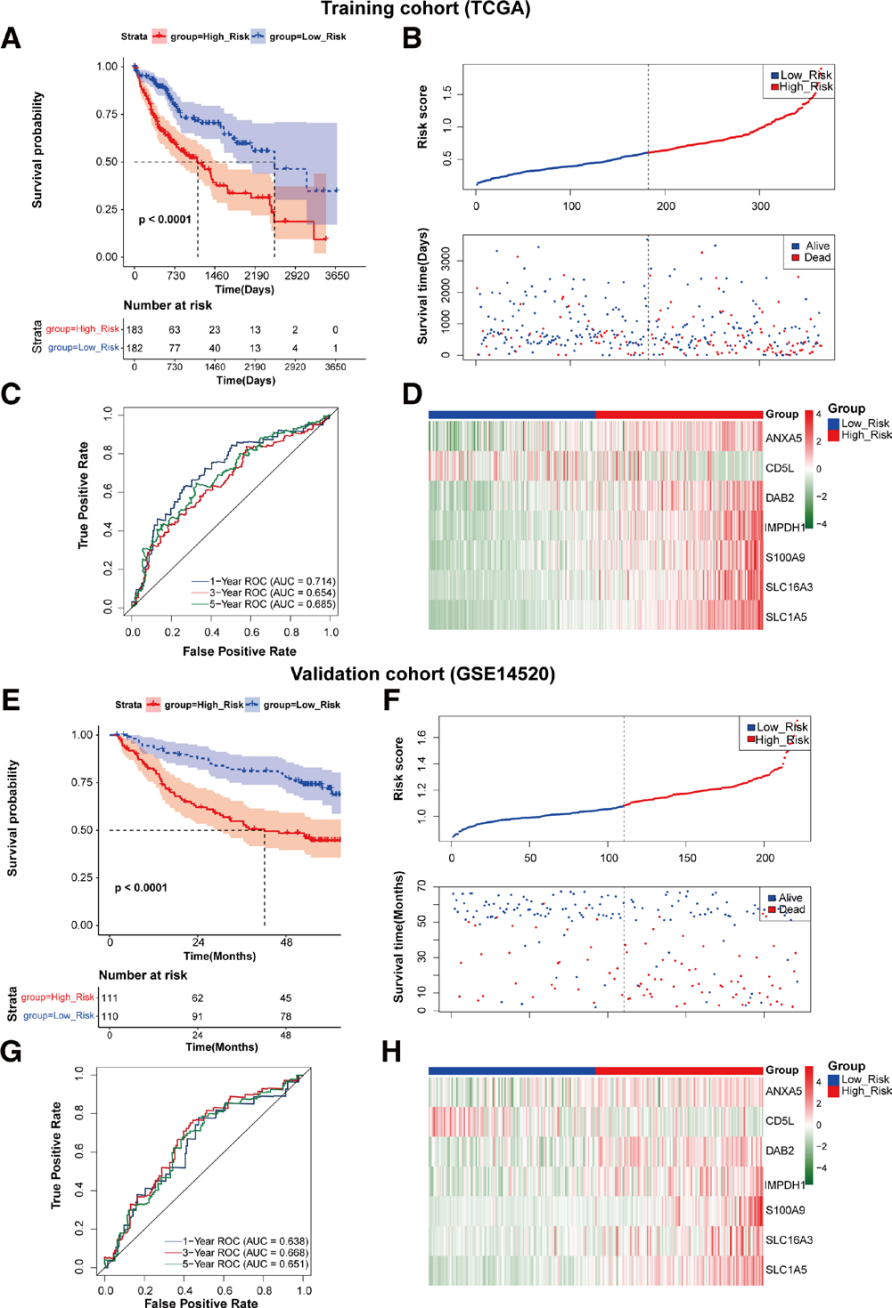
Evaluation of the prognostic performance of established model in the training and validation sets. (A and E) KM curves demonstrated the survival of patients in the low- and high-risk groups in the TCGA (A) and GSE14520 (E). (B and F) Distributions of RS value and survival status of patients in TCGA (B) and GSE14520 (F). (C and G) ROC curves revealed the predictive efficacy of 1-, 3-, and 5-yr overall survival in TCGA (C) and GSE14520 (G). (D and H) Heatmap of 7 survival-related genes expression patterns in low- or high-risk groups in TCGA (D) and GSE14520 (H). KM = Kaplan–Meier, ROC = receiver operator characteristics, TCGA = the cancer genome atlas.

Using a univariate Cox analysis, we found that 26 genes were considered as prognostic factors (Fig. 8A). Then, Lasso Cox regression was adopted to eliminate redundancy and finally the prognostic model composed of 7 genes (ANXA5, CD5L, DAB2, IMPDH1, S100A9, SLC16A3, and SLC1A5) was established (Fig. 8B and C). The RS of this model was calculated as follows: RS = (0.000156327 × Exp [ANXA5]) + (−0.040423996 × Exp [CD5L]) + (0.02125259 × Exp [DAB2]) + (0.03337938 × Exp [IMPDH1]) + (0.056557488 × Exp [S100A9]) + (0.035289223 × Exp [SLC16A3]) + (0.143756984 × Exp [SLC1A5]). Based on the median cutoff threshold, all patients in the TCGA cohort were divided into 2 risk subgroups: LR and HR. KM curve displayed that patients in the HR group were significantly associated with shorter survival time (*P* < .0001, Fig. 9A). The distribution of RS and survival status of patients is shown in Figure 9B. It can be observed that the patients in the HR group had a higher probability of death than those in the LR group (Fig. 9C). In addition, ROC curves suggested that AUC (areas under the ROC) values for 1-, 3-, and 5-year were 0.714, 0.656, and 0.685, respectively. The expression pattern of prognostic signature is displayed in Figure 9D. In brief, patients in the HR group had elevated expression of ANXA5, DAB2, IMPDH1, S100A9, SLC16A3, and SLC1A5, while had decreased expression of CD5L. Further, the predictive performance of established model was also validated using a GEO dataset (GSE14520). Patients were classified into LR and HR groups based on the median RS. Similar to the results in the training set, patients with a HR score had a considerably increased risk of mortality (Fig. 9E). The survival status of patients with HCC are shown in Figure 9F, and more deaths were observed in the HR group. The AUC values in the validation cohorts were 0.638, 0.668, and 0.651 at 1-, 3-, and 5-year, respectively (Fig. 9G). The gene expression patterns of patients in the different risk groups are displayed in Figure 9H, which was consistent with those in the training cohort.

## 4. Discussion

We found that LRRC1 was upregulated in HCC tissues at the transcriptional and protein levels. Abundant LRRC1 expression in HCC indicates a poor prognosis. The DEGs between the LRRC1-high and -low groups were significantly enriched in pathways associated with cancer, amino acid metabolism, carbohydrate metabolism, and the immune system. We identified 15 differentially infiltrated immune cells between the LRRC1-high and -low groups and found that LRRC1 expression correlated with 14 of them, including macrophages, Tregs, and NK cells. Based on the differentially infiltrated immune cells, we used WGCNA to identified 83 immune cell-related genes, of which 27 were prognosis-related. A PPI network revealed that ANXA5, MMP9, and NCF2 were hub genes. Transcription factor CREB1 regulated ANXA5, MMP9, and LRRC1 in the TF regulatory network. Further, a 7-gene signature was identified. The median RS calculated by this model was used to divide HCC samples to 2 risk groups, and the HCC patients with HR score had a shorter overall survival.

The cell polarity regulator proteins drosophila disc large tumor suppressor 1 and post-synaptic density protein-95 and LRRC1 interact on the basolateral side of human epithelial cells.^[19]^ Loss of epithelial cell polarity has been implicated in liver cancer progression.^[38]^ Thus, the idea that LRRC1 is aberrantly overexpressed in HCC tissues and cell lines compared to normal controls is reasonable,^[11]^ which further supports our findings, that is, LRRC1 was upregulated in HCC tissues at the transcriptional and protein levels. Moreover, our study also indicated that the expression level of LRRC1 varied across different stages or grades. However, fewer data were available on the contribution of LRRC1 to cancer development. Evidence suggests that LRRC1 is located on chromosome 6p12.1, a region frequently amplified in HCC^[11]^; the overexpression of LRRC1 is associated with its mislocalization from the plasma membrane to the cytoplasm, possibly due to the activation of the AKT/ATF2 pathway and loss of membranous E-cadherin.^[38]^ Together, these findings shed a certain degree of light on the molecular mechanisms by which LRRC1 drives HCC. However, the precise contribution of LRRC1 to the different stages of the tumor remains to be further investigated. Moreover, we observed that high LRRC1 expression indicated low rates of disease-free survival, overall survival, and a poor clinical prognosis. Thus, LRRC1 might be a risk factor for HCC.

We observed that DEGs between high- and low- LRRC1 expression groups were significantly enriched in pathways associated with cancer, amino acid metabolism, carbohydrate metabolism, cell growth and death, and the immune system. Accumulated evidence indicates that interference with cellular metabolism predisposes humans to cancer. Amino acids are important in driving nucleoside synthesis, maintaining cellular redox homeostasis, and generating energy. Therefore, an abundant supply of amino acids is necessary for cancer cells to maintain their proliferative drive.^[39]^ In addition to amino acid metabolism, carbohydrate metabolism also plays a crucial role in tumor progression.^[40]^ Rates of aerobic glycolysis are high in many human cancers.^[41]^ Increased glucose metabolism enhances lipogenesis and nucleotide biosynthesis and promotes tumor cell proliferation by providing essential bioenergy molecules.^[42,43]^ LRRC1 has been shown to regulate the WNT/β-catenin signaling pathway in breast cancer stem cells, which is a key hub for modulating lipogenic/osteogenic differentiation of bone marrow mesenchymal stem cells.^[44,45]^ In addition, recent proteomic analysis has revealed that LRRC1 acts as a downstream target of PPARγ (a transcription factor essential for adipogenesis regulation) to regulate the expression of adipogenesis-relate proteins, such as FASN and SCD.^[46]^ However, the detailed role of LRRC1 in glycolipid metabolism has not been reported and will be explored in our future studies. Taken together, increased LRRC1 expression may promote HCC progression by regulating amino acid and carbohydrate metabolism.^[40]^

Mutations in TP53 and CTNNB1 are considered drivers of HCC development.^[47]^ Wild-type p53 protein plays a key role in regulation of the cell cycle and apoptosis after DNA damage.^[48]^ Cells with p53 mutations can escape apoptosis and transform into cancer cells after DNA damage.^[49]^ A TP53 mutation is the most common genetic change in HCC, with an average mutation frequency of 30%,^[48]^ that can increase to 60% when HCC is associated with the hepatitis B virus.^[50]^ Mutations in TP53 correlate with tumor differentiation, vascular invasion, and tumor stage in HCC,^[48]^ and the most frequently mutated oncogene in HCC, CTNNB1, correlates with elevated glutamine synthetase levels and vascular invasion.^[51]^ Mutations in TP53 and CTNNB1 appear to be mutually exclusive.^[47]^ The present findings found that the mutation frequencies of TP53 were 42% and 18%, and those of CTNNB1 were 13% and 37% in the LRRC1-high and -low groups, respectively. These findings suggested that mutation landscapes differ between HCC with high and low LRRC1 expression.

Immune cell infiltration plays a critical role in tumorigenesis.^[52]^ The present study revealed 15 differentially infiltrated immune cells between the LRRC1-high and -low groups, 14 of which were significantly associated with LRRC1 expression. For instance, macrophages and Treg cells positively correlated with LRRC1 expression. Due to their roles in immune invasion, increased numbers of tumor-associated Tregs and macrophages are associated with a poor prognosis for patients with HCC.^[53,54]^ In addition, we observed that LRRC1 expression was negative correlated with NK cell infiltration. NK cells are innate lymphoid cells that can inhibit the invasion of a variety of tumor cells by releasing perforin/granzyme, secreting cytokines (such as TNF-α), and activating apoptotic pathways.^[55]^ Meanwhile, It has been proven that activated NK cells are associated with better prognosis for HCC patients.^[56]^ Hence, NK cells have great potential in the immunotherapy of HCC.^[57]^ Besides, in our results, the DEGs between the LRRC1-high and -low groups were significantly enriched in pathways associated with the immune system.

Therefore, attenuated antitumor immunity could explain the poor prognosis of patients in the LRRC1-high group, and LRRC1 could be a potential immune-associated prognostic biomarker for HCC.

In this study, PPI network revealed that ANXA5, MMP9, and NCF2 were hub genes. The TF CREB1 regulated ANXA5, MMP9, and LRRC1 in the TF regulatory network. The overexpression of ANXA5, which is a calcium-dependent phospholipid binding protein, potentially facilitates lymphatic metastasis of HCC.^[58]^ This protein has been applied as an immune checkpoint inhibitor in cancer treatment.^[59]^ MMP9 in the MMP family participates in inflammation, immunity, wound repair, and embryonic development under normal conditions. Under external stimulation, MMP9 can degrade the extracellular matrix of local tissues to promote tumor invasion and metastasis.^[60]^ The CREB1 of the leucine zipper family is essential for DNA repair, cell cycle, and apoptosis.^[61]^ Once activated, CREB1 can regulate its downstream target genes, including MMP9^[62]^; this result is consistent with ours. The regulatory relationship between CREB1 and LRRC1 has not been reported to the best of our knowledge. Given that ANXA5 and MMP9 were identified as DEGs in the LRRC1-high and -low groups, we speculated that a regulatory mechanism functions between LRRC1 and the CREB1-MMP9/ANXA5 axis during the immune response to HCC. Further studies are needed to understand the underlying mechanisms.

Following, we constructed a prognostic model by performing univariate and Lasso Cox regression analysis on 27 prognostic genes. The model could divide HCC patients into HR and LR groups. KM survival curves showed that HCC patients with HR scores had extremely poor prognosis, but those with LR score had better life expectancy, indicating that the model had a strong prognostic predictive potential. This model included ANXA5, CD5L, DAB2, IMPDH1, S100A9, SLC16A3, and SLC1A5. CD5L presented with abnormal mRNA and protein levels in HCC and it was found to be an independent prognostic factor for HCC patients.^[63,64]^ DAB2 promotes epithelial-mesenchymal transition and invasion of tumor cells at advanced tumor stages, and it acts as a risk factor in HCC,^[65]^ which is consistent with our findings. High expression of IMPDH1 is associated with poor prognosis of HCC patients and may serve as a potential target for immunotherapy.^[66]^ S100A9 is a key oncogene in HCC progression, and its expression level is negatively correlated with the prognosis of patients.^[67]^ Moreover, SLC16A3, and SLC1A5 have also been confirmed to be prognostic targets in HCC.^[68,69]^ Taken together, this model is valuable in guiding the prognosis prediction and molecular targeted therapy of HCC.

This study is the first comprehensive and detailed analysis of the role of LRRC1 in HCC, and suggests that LRRC1 as a potential prognostic and diagnostic biomarker for HCC by elucidating its expression, prognostic value, association with clinicopathological factors and immune infiltration in HCC. However, the shortcomings of this study are as follows: our results were obtained through bioinformatics analysis but were not validated in vitro or in vivo; although the mRNA and protein levels of LRRC1 were different between HCC and normal tissues, the mechanism of LRRC1 in HCC progression remains to be further investigated. In the future, we will conduct more in-depth research on this topic.

In conclusion, our findings suggested that LRRC1 expression is upregulated in HCC tissues, and that upregulated LRRC1 expression indicates a poor prognosis for patients with HCC. The DEGs between LRRC1-high and -low groups were associated with the immune system, suggesting that LRRC1 could be a potential immune-associated prognostic biomarker of HCC.

## Author contributions

**Conceptualization:** Qingshan Cai.

**Data curation:** Dongyang Wu, Yueling Shen.

**Formal analysis:** Shudong Li.

**Investigation:** Qingshan Cai, Yueling Shen, Liyou Liu, Limin Wang.

**Methodology:** Dongyang Wu, Yueling Shen, Shudong Li, Xiaonan Chen, Limin Wang.

**Project administration:** Jianxing Zheng.

**Resources:** Dongyang Wu, Dong Liu.

**Software:** Liyou Liu, Dong Liu, Yong Li.

**Supervision:** Jianxing Zheng, Xiaonan Chen.

**Validation:** Shudong Li, Dong Liu.

**Visualization:** Yong Li.**Writing – original draft:** Qingshan Cai.

## Supplementary Material


