# Supplementary figures and images for: Prognostic significance of LRRC1 in hepatocellular carcinoma and construction of relevant prognostic model
Source: Medicine (Baltimore). 2023 Jul 28;102(30):e34365. doi: 10.1097/MD.0000000000034365 (PMC10378736; doi:10.1097/MD.0000000000034365)

**Supplemental Figure S1.** Analysis of genes associated with survival.

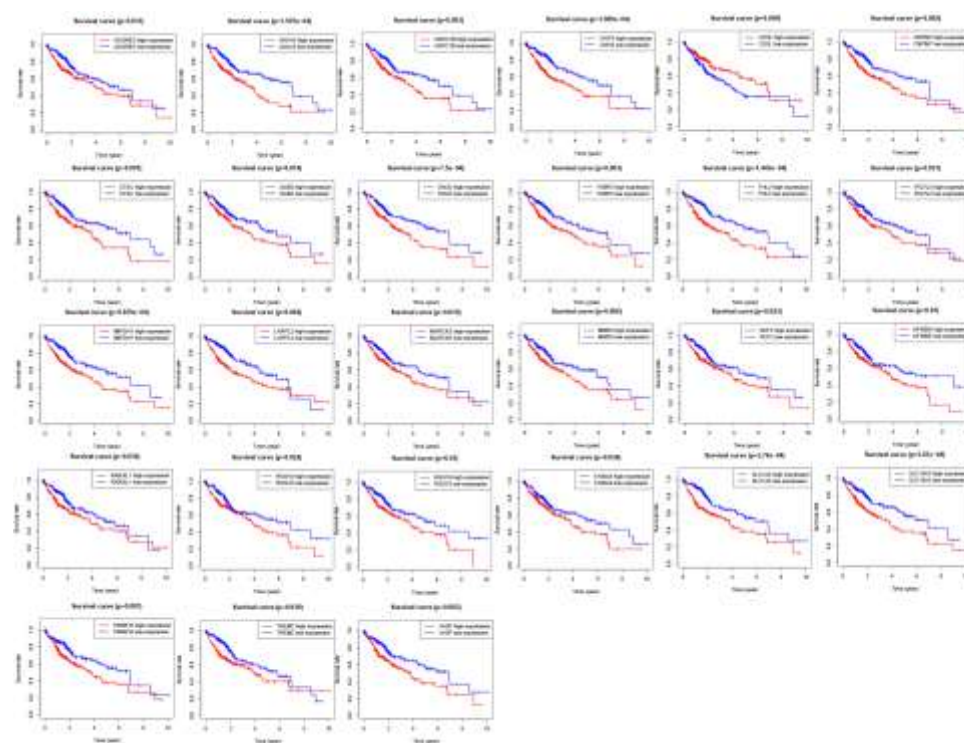

Supplement: Supplementary file 1 [file medi-102-e34365-s001.pdf]
